# Immunomodulatory Effects of Hydroxychloroquine and Chloroquine in Viral Infections and Their Potential Application in Retinal Gene Therapy

**DOI:** 10.3390/ijms21144972

**Published:** 2020-07-14

**Authors:** Laurel C. Chandler, Imran H. Yusuf, Michelle E. McClements, Alun R. Barnard, Robert E. MacLaren, Kanmin Xue

**Affiliations:** 1 Nuffield Laboratory of Ophthalmology, Nuffield Department of Clinical Neurosciences & NIHR Oxford Biomedical Research Centre, University of Oxford, Oxford OX3 9DU, UK; imran.yusuf@merton.ox.ac.uk (I.H.Y.); michelle.mcclements@eye.ox.ac.uk (M.E.M.); alun.barnard@eye.ox.ac.uk (A.R.B.); maclaren@eye.ox.ac.uk (R.E.M.); 2 Oxford Eye Hospital, Oxford University Hospitals NHS Foundation Trust, Oxford OX3 9DU, UK

**Keywords:** hydroxychloroquine, chloroquine, adeno-associated virus, AAV, gene therapy, innate immunity, TLR9, cGAS, SARS-CoV-2

## Abstract

Effective treatment of retinal diseases with adeno-associated virus (AAV)-mediated gene therapy is highly dependent on the proportion of successfully transduced cells. However, due to inflammatory reactions at high vector doses, adjunctive treatment may be necessary to enhance the therapeutic outcome. Hydroxychloroquine and chloroquine are anti-malarial drugs that have been successfully used in the treatment of autoimmune diseases. Evidence suggests that at high concentrations, hydroxychloroquine and chloroquine can impact viral infection and replication by increasing endosomal and lysosomal pH. This effect has led to investigations into the potential benefits of these drugs in the treatment of viral infections, including human immunodeficiency virus and severe acute respiratory syndrome coronavirus-2. However, at lower concentrations, hydroxychloroquine and chloroquine appear to exert immunomodulatory effects by inhibiting nucleic acid sensors, including toll-like receptor 9 and cyclic GMP-AMP synthase. This dose-dependent effect on their mechanism of action supports observations of increased viral infections associated with lower drug doses. In this review, we explore the immunomodulatory activity of hydroxychloroquine and chloroquine, their impact on viral infections, and their potential to improve the efficacy and safety of retinal gene therapy by reducing AAV-induced immune responses. The safety and practicalities of delivering hydroxychloroquine into the retina will also be discussed.

## 1. Introduction

The 4-aminoquinoline anti-malarial drugs, hydroxychloroquine and chloroquine, have been frequently used in the treatment of autoimmune diseases such as systemic lupus erythematosus and rheumatoid arthritis. Their utility in dampening these autoimmune diseases provides insights into their immunomodulatory activities [1]. Hydroxychloroquine and chloroquine are weak bases that accumulate within acidic intracellular compartments, such as lysosomes [2]. When used at high concentrations, hydroxychloroquine and chloroquine appear to lead to a dose-dependent increase in endosomal and lysosomal pH [3,4]. In contrast, low concentrations of hydroxychloroquine and chloroquine may exert a separate immunomodulatory effect on intracellular innate immune responses to viral infections, with minimal interference in lysosomal activity [5,6].

Adeno-associated viruses (AAV) are the most commonly used viral vectors for gene therapy due to their broad tissue tropism, relatively low immunogenicity, and lack of pathogenicity. AAV are non-replicating, non-enveloped parvoviruses with a single-stranded DNA (ssDNA) genome. A recombinant AAV transgene typically contains a therapeutic gene expression cassette driven by a ubiquitous or tissue-specific promoter with a generic polyadenylation signal. The cassette is flanked by viral inverted terminal repeats (ITRs), which aid persistence through the formation of concatemeric episomes within the host cell nucleus [7]. The episomes can sustain long-term transgene expression while minimising the potential for mutagenesis. Nonetheless, AAV-specific immune responses have been observed, which may induce potentially harmful inflammatory reactions and compromise the outcome of gene therapy [8].

AAV-mediated gene therapies have been successfully applied to a number of monogenic retinal dystrophies, including *RPE65*-associated Leber congenital amaurosis which has received Food and Drug Administration (FDA) approval, and choroideremia and *RPGR*-associated X-linked retinitis pigmentosa which are in advanced clinical trials [9,10,11,12,13,14]. Despite promising results in clinical trials, dose-dependent immunogenicity of AAV has been observed following retinal gene therapy, requiring high-dose immunosuppression in some cases [14,15,16,17]. There is thus interest in adjuvant therapies, either local or systemic, to reduce inflammatory responses and improve the long-term durability of gene therapies.

In this review, we discuss the immunomodulatory effects of hydroxychloroquine and chloroquine and how these might be relevant in the treatment of viral infections. We will then discuss how hydroxychloroquine might be used to control immune responses to AAV and enhance the efficacy of gene therapy.

## 2. Immunomodulatory Mechanisms of Hydroxychloroquine and Chloroquine

Hydroxychloroquine and chloroquine have been used for many decades in the treatment of a wide range of infections, including malaria (prozozoal), Q fever (bacterial), and human immunodeficiency virus (HIV) (viral), and autoimmune diseases such as systemic lupus erythematosus and rheumatoid arthritis [18]. Despite the proposal of numerous putative modes of action involving interactions with a range of molecular targets and signalling pathways, the immunomodulatory effects of hydroxychloroquine and chloroquine remain uncertain.

### 2.1. Effects on Intracellular Innate Immune Responses

**Figure 1:**
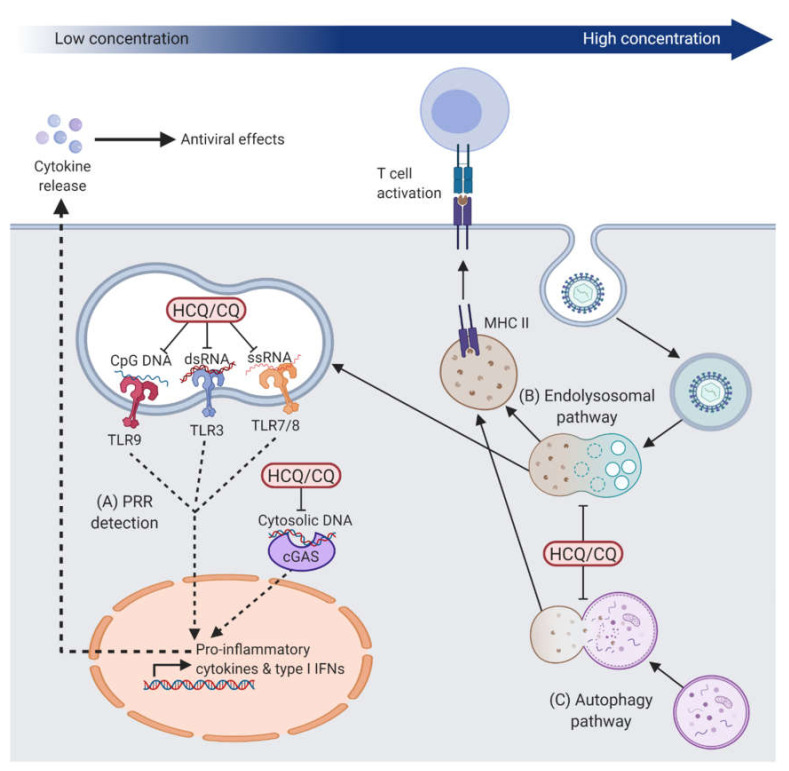
Immunomodulatory mechanisms of hydroxychloroquine (HCQ) and chloroquine (CQ). (**A**) At lower concentrations, which we define as ≤20 μM, HCQ and CQ can inhibit the activation of nucleic acid sensors, Toll-like receptors (TLR) in endosomes and cyclic GMP-AMP synthase (cGAS) in the cytoplasm. This leads to the inhibition of pattern recognition receptor (PRR)-induced activation of downstream pro-inflammatory cytokine and type I interferon (IFN) gene expression. At high concentrations (≥100 μM), HCQ and CQ can increase lysosomal pH, which leads to disruption of presentation of (**B**) extracellular antigens processed through the endolysosomal pathway and (**C**) intracellular antigens processed through the autophagosome-lysosome fusion pathway by antigen presenting cells. dsRNA, double-stranded RNA; ssRNA; single-stranded RNA; MHC II, major histocompatibility complex class II.

A major advance in the effort to understand the mechanism of action of hydroxychloroquine and chloroquine was the discovery of their antagonistic effects on members of the toll-like receptor (TLR) family, a group of pattern recognition receptors (PRRs) that can detect molecular signatures of pathogens, such as bacteria and viruses, in order to elicit rapid intracellular immune responses [19]. Within this family, TLR3, TLR7, TLR8, and TLR9 are nucleic acid sensors that are exclusively found within endosomes so to minimise exposure to self-DNA or –RNA [20]. TLR3 detects double-stranded RNA (dsRNA) [21], TLR7 and TLR8 recognise single-stranded RNA (ssRNA) [22], and TLR9 is activated by DNA containing unmethylated CpGs which are abundant in microbial but rare in vertebrate genomes (i.e., 60–90% of mammalian CpGs are methylated) [23,24]. These nucleic acid ligands are also common features of viral genomes, thus TLRs play an important role in the detection of viruses within cells as part of the innate immune defence against viral infections. Initial in vitro studies demonstrated that chloroquine could suppress the stimulatory activity of CpG-oligodeoxynucleotides (ODNs) at concentrations ≤10 μM, thereby inhibiting cytokine release [5,25]. Subsequent studies demonstrated the dose-dependent inhibition of CpG-ODN binding to TLR9 by ≤2.5 μM chloroquine in human embryonic kidney 293 (HEK293) cells [26] (Figure 1). Although this interaction was optimal in acidic endosomal environments at pH 5.5–6.5 [26], TLR9 inhibition can be achieved with significantly lower concentrations of chloroquine than that needed to alter the pH of endosomal compartments (≤20 μM) [4,5,6]. Based on these findings, it was proposed that hydroxychloroquine and chloroquine could bind to CpG-containing nucleic acids and induce conformational changes that prevent them from interacting with and activating TLR9 [6,27]. Furthermore, chloroquine inhibited ssRNA-mediated TLR8 activation and poly(I:C) (synthetic analogue of dsRNA)-mediated TLR3 activation in HEK293 cells in a dose-dependent manner (≤20 μM) [6]. In human monocyte-derived dendritic cells and peripheral blood mononuclear cells (PBMCs), chloroquine inhibited poly(I:C)-induced TLR3 responses at concentrations ≤10 μM [28]. Chloroquine has also been shown to inhibit TLR7-mediated responses to small nuclear ssRNA in human PBMCs at a concentration ≤10 μM, resulting in the downregulation of IFN-α expression [29] (Figure 1).

In addition to the inhibition of TLRs, hydroxychloroquine has been shown to have an inhibitory effect on the PRR cyclic GMP-AMP synthase (cGAS). cGAS is activated by cytosolic DNA, which is indicative of viral infection, to initiate a signalling cascade leading to a type I interferon response [30,31]. An in silico screen of drug libraries to find cGAS antagonists identified several antimalarial drugs as putative inhibitors and further computational analysis predicted an interaction between hydroxychloroquine and the DNA binding site of cGAS [32]. Following dsDNA transfection of the human monocytic cell line (THP1), hydroxychloroquine treatment decreased expression of interferon (IFN)-β, a downstream cytokine of cGAS activation, in a dose-dependent manner with a half-maximal inhibitory concentration (IC50) of 25 μM [32] (Figure 1). It remains unclear whether the inhibitory effect of hydroxychloroquine on cGAS is achieved through hydroxychloroquine binding to cGAS or the DNA. Nonetheless, given the proposed mechanism of action via TLR9, it seems possible that hydroxychloroquine and chloroquine might prevent the activation of nucleic acid sensors cGAS and TLR9 through a common mechanism such as alteration of the structural configuration of the DNA substrate [33,34].

These findings have gone some way to explain the efficacy of hydroxychloroquine and chloroquine in the treatment of systemic lupus erythematosus and rheumatoid arthritis. A hallmark of both autoimmune diseases is the accumulation of DNA- or RNA-containing complexes due to a build-up of apoptotic bodies, which have been implicated in the activation of TLR9 or TLR7, respectively [35,36]. Recent studies in patient-derived PBMCs have suggested that cGAS may also be important in the detection of DNA in systemic lupus erythematosus [37,38]. Indeed, redundancy and overlap between the substrates of different nucleic acid sensors seem likely and evolutionarily advantageous. In preventing the activation of these PRRs, hydroxychloroquine or chloroquine may be able to dampen an overactive innate immune response to self-nucleic acids in systemic lupus erythematosus and rheumatoid arthritis. The same mechanism would also enable hydroxychloroquine and chloroquine to reduce the immune response to foreign nucleic acid antigens, such as those derived from viral infections.

### 2.2. Effects on Cellular Immune Responses

**Figure 2:**
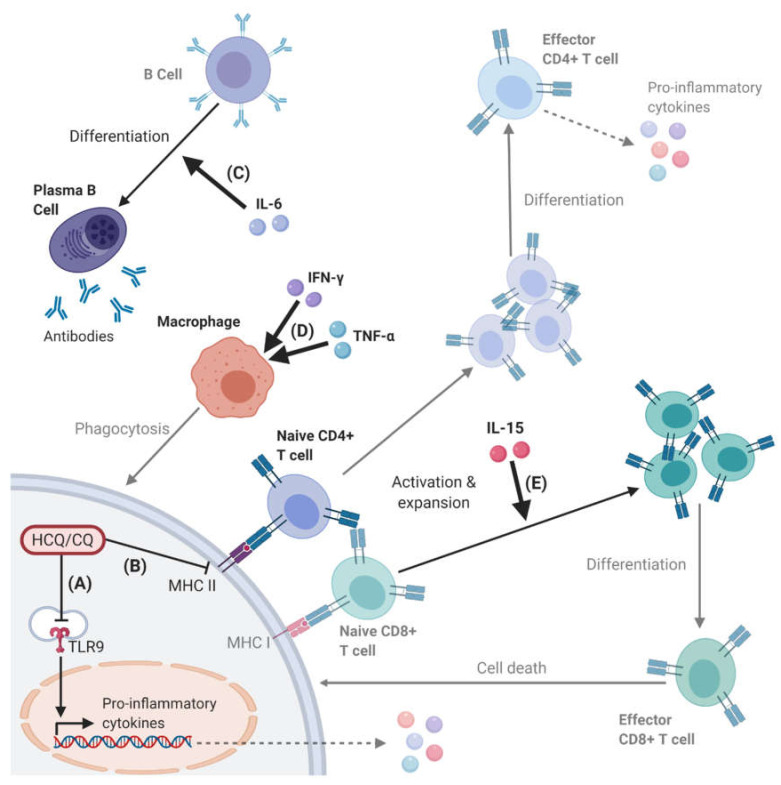
Effects of pro-inflammatory cytokines on cell-mediated immune responses. An example of cell-mediated immune responses downstream of the immunomodulatory effects of HCQ and CQ. HCQ and CQ can reduce pro-inflammatory cytokine expression by inhibition of (**A**) PRRs (e.g., TLR9) at low concentrations or (**B**) MHC II-mediated antigen presentation at high concentrations (see Figure 1). MHC II can activate CD4^+^ T cells, which are another major source of pro-inflammatory cytokines. Key pro-inflammatory cytokines include (**C**) interleukin (IL)-6 that can stimulate the maturation and expansion of B cells into antibody-producing plasma cells, (**D**) IFN-γ and tumour necrosis factor-α (TNF-α) that can activate macrophages, and (**E**) IL-15 that can stimulate the activation and expansion of CD8^+^ T cells. Lower opacity text and images represent the indirect effects, while those in bold highlight the direct effects of HCQ, CQ, and pro-inflammatory cytokines.

The inhibition of key PRRs, such as TLR9 and cGAS, by hydroxychloroquine and chloroquine has been shown to affect downstream pro-inflammatory cytokine and type I interferon responses (Figure 2) [6,29,32]. In vitro, both hydroxychloroquine and chloroquine have been shown to inhibit the production of the pro-inflammatory cytokines tumour necrosis factor-α (TNF-α), IFN-γ and interleukin (IL)-6 in PBMCs [39], and TNF-α, IL-1β, and IL-6 in human monocytes [40]. IFN-γ can prime macrophages for pro-inflammatory responses and render them resistant to suppressive immune factors [41]; IL-6 can control the survival, expansion, and maturation of B cells [42]; and TNFα is key in activating and promoting the long-term survival of macrophages [43]. Chloroquine has also been demonstrated to inhibit c-Jun N-terminal kinase (JNK)-mediated activation of the transcription factor activator protein 1 (AP-1) in CD4^+^ T cells [44]. The resulting reduction in helper T cell activation, proliferation, and cytokine release would be expected to reduce the level of immune response. A type I interferon response also induces an anti-viral state within the infected and neighbouring cells by upregulating a large number of interferon-stimulated genes (ISGs) via the Janus kinase-signal transducer and activator of transcription (JAK-STAT) pathway [45]. Examples include tripartite motif-containing 5α (TRIM5α) that can bind to viral capsid proteins to promote viral disassembly and restrict infection [45]; ISG15 which is a ubiquitin-like protein that can covalently bind to viral proteins to disrupt replication [46]; and IL-15 that helps to eliminate infected cells by inducing the proliferation and maintenance of natural killer cells and CD8^+^ T cells [47,48].

In addition to the inhibition of PRRs and their downstream signalling cascades, hydroxychloroquine and chloroquine also appear capable of indirectly modulating immune responses though the alkalisation of lysosomes when used at higher concentrations. The processing of extracellular material for antigen presentation is classically undertaken by professional antigen-presenting cells, which utilise the lysosomal pathway to display short peptides on major histocompatibility complex (MHC) class II molecules for detection by CD4^+^ T cells [49]. By increasing the pH of lysosomes, chloroquine can affect antigen processing, as demonstrated in macrophages (at 100 μM) [50], dendritic cells (at 100 μM) [51] and B cells (at 25–100 μM) [52]. Notably, these effects, which are associated with raised endosomal and lysosomal pH, were primarily observed at high concentrations of chloroquine (100 μM). In addition, chloroquine has been shown to inhibit autophagy, a regulated process for removing damaged intracellular organelles or proteins by enveloping cytoplasmic material, by inhibiting the fusion between autophagosomes and lysosomes [53]. This, in turn, leads to the inhibition of presentation of cytosolic antigens, including autoantigens and viral antigens, on MHC class II molecules via the autophagy-lysosomal pathway [54,55,56] (Figure 1). Since dysregulation of autophagy contributes to cancer development and chemotherapy resistance, hydroxychloroquine has been evaluated as an autophagy inhibitor in clinical trials for various cancers [57,58,59,60]. Very high daily doses of hydroxychloroquine (typically ≤1200 mg/day) have been used in this context in order to effectively block the fusion of lysosomes with autophagosomes, thus supporting a dose-dependent mechanism of action of hydroxychloroquine. Overall, study findings are consistent with hydroxychloroquine and chloroquine having a direct immunosuppressive effect on viral sensing and pro-inflammatory cytokine release at low concentrations, and an indirect immunomodulatory effect via lysosomal antigen processing and presentation at high concentrations (Figure 1).

## 3. Effects of Hydroxychloroquine and Chloroquine on Viral Infections

Historically, hydroxychloroquine and chloroquine have been utilised as tools to assess the effect of endosomal and lysosomal processes on viral entry, as many viruses exploit the host endocytic machinery to infect target cells. Following cell surface receptor binding, viral particles are internalised via a range of endocytic mechanisms, including clathrin-mediated, caveolin-mediated, and non-clathrin non-caveolin endocytosis. Endosomes undergo gradual acidification from an early to late stage, which acts as a trigger for viral uncoating and genome release so that endosomal escape is precisely timed to prevent overexposure of viral contents to the acidic environment [61]. The inhibition of replication of Semliki Forest virus [62], Chikungunya virus [63], and hepatitis A virus [64] by chloroquine in vitro has been suggested to occur as a result of impaired endosomal acidification and viral entry due to the concentration of this drug in intracellular compartments. In addition, the acidification of vesicular compartments along the secretory pathway is important in the post-translational modification of viral envelope proteins during the production of new infectious virions. For example, in vitro studies demonstrated that hydroxychloroquine and chloroquine could inhibit the production of infectious HIV-1 virions by preventing glycosylation of the viral glycoprotein gp120, which normally occurs within the acidic Golgi complex [65,66,67]. In addition, chloroquine treatment resulted in the accumulation of non-infectious herpes simplex virus (HSV)-1 virions in the trans-Golgi network in vitro [68]. Taken together, these observations support the potential anti-viral effects of hydroxychloroquine and chloroquine in vitro when used at sufficient concentrations to alter endosomal pH, but in vivo efficacy remains unclear.

### 3.1. Immunomodulatory Effects on Viral Infections

The combination of the alkalisation of the endolysosomal pathway and the immunomodulatory effects of hydroxychloroquine and chloroquine have led to speculations surrounding their potential benefits in the treatment of viral infections. Although both innate and adaptive immune responses are vital to recovery from viral infections, over activation of immune responses may lead to harmful tissue damage and disease progression. Such counterproductive immune responses have been observed in chronic HIV [69], severe acute respiratory syndrome coronavirus (SARS-CoV) [70], and SARS-CoV-2 infections [71], leading to investigation of hydroxychloroquine and chloroquine as potential immunomodulatory therapies. Systemic T cell activation in chronic HIV infection is thought to be associated with disease progression. A randomised controlled trial showed that chloroquine therapy could reduce the proportion of CD38^+^ HLA-DR^+^ CD8 T cells, thus supporting its usage in certain groups of HIV patients [72]. The effect may be explained by chloroquine-mediated suppression of TLR7 activation by HIV RNA in primary monocytes and plasmacytoid dendritic cells (pDCs) [73,74,75]. Similarly, in vitro chloroquine has been shown to prevent TLR7-mediated responses to other RNA viruses such as hepatitis C [76,77], influenza [78] and vesicular somatic virus [79], as well as TLR9-mediated responses to DNA viruses such as HSV-2 and Epstein–Barr virus [80,81,82]. There appears to be a general correlation between the concentration of chloroquine or hydroxychloroquine used and the effects seen in vitro (Table 1). High doses of these drugs, which we define as ≥100 μM, tend to inhibit viral replication, while low doses (≤20 μM) tend to inhibit innate immune pathways without detectable effect on viral replication. This correlation is consistent with the hypothesis that high concentrations of hydroxychloroquine or chloroquine could inhibit viral entry or assembly by increasing the pH of intracellular vesicular compartments, while low concentrations could reduce pro-inflammatory cytokine release by preventing the activation of nucleic acid sensors. Nonetheless, a degree of overlap could be expected between these two types of effects, which may be variable between different cell lines. Of note, Vero cells derived from the kidney epithelium of the African green monkey, which are commonly used to propagate viruses in vitro, harbour a 9 Mb deletion leading to loss of the type I interferon gene cluster [83,84]. Therefore, Vero cells would not be an appropriate model for assessing the immunomodulatory effects of these drugs during viral infection. Further work is needed to assess the effects of hydroxychloroquine and chloroquine on viral infection in biologically relevant cell types and in vivo.

In contrast to their inhibitory effects on viral replication in vitro, hydroxychloroquine and chloroquine may have different effects in vivo due to the lower systemic concentrations achieved and their inhibitory effects on anti-viral responses, such as those mediated through TLRs. In a randomised, double-blind, controlled trial of 400 mg/day of hydroxychloroquine versus placebo in patients with asymptomatic HIV infection and not on anti-retroviral therapy, hydroxychloroquine was found to be associated with an increased HIV viral RNA load as well as a faster decline in CD4^+^ cell counts, leading to the need to initiate anti-retroviral therapy [87]. Another double-blind controlled trial of HIV-1 infected patients randomised to either 250 mg/day of chloroquine or placebo, demonstrated a significant increase in HIV viral RNA in patients not taking anti-retroviral therapy and a decrease in ISG expression [88]. While in vitro experiments in Vero cells showed chloroquine could inhibit the replication of Semliki Forest virus and the closely related Chikungunya virus, these effects were not seen in animal studies where chloroquine was associated with higher viral loads [89,90]. In addition, when used to treat Chikungunya viral infection in a clinical trial, chloroquine led to clinical deterioration without significant effect on viral load, which was attributed to a delaying effect on adaptive immunity [90,91].

### 3.2. Treatment of SARS-CoV-2 Infections

The anti-viral applications of hydroxychloroquine and chloroquine have gained renewed interest in the wake of the 2020 SARS-CoV-2 pandemic. In vitro studies of SARS-CoV, responsible for the 2003 epidemic, demonstrated that chloroquine had an inhibitory effect on viral replication in Vero cells [92,93,94]. However, intraperitoneal and intranasal injections of chloroquine ultimately induced no effect in vivo in BALB/c mice [95]. Nevertheless, with the emergence of the SARS-CoV-2 global pandemic, several in vitro studies have demonstrated hydroxychloroquine and chloroquine inhibit viral replication of this new strain of coronavirus in Vero cells [96,97,98]. However, the potential benefits of these drugs in the treatment of SARS-CoV-2 infected patients are highly disputed. Preliminary clinical trials in China claimed that chloroquine treatment might inhibit exacerbation of pneumonia, improve radiographic appearances, enhance virus-negative conversion, and reduce the overall length of the illness [99]. However, the results of these trials are yet to be formally peer-reviewed. A subsequent French trial with 20 SARS-CoV-2 patients suggested that hydroxychloroquine treatment (at 600 mg daily) significantly reduced viral load [100]. However, methodological concerns, such as a lack of randomisation and unexplained exclusion of patients in the hydroxychloroquine-treated arm (three of which were transferred to intensive care and another died), have since led to the discounting of the study’s findings [101]. In a subsequent larger double-blind clinical trial, 81 SARS-CoV-2 infected patients randomised to either low (450 mg twice daily on the first day and once daily for four days) or high dose (600 mg twice daily for 10 days) chloroquine were compared, with both groups also receiving the antibiotic azithromycin. Increased lethality from cardiac effects associated with a prolongation of the corrected QT interval was observed on electrocardiography in the high dose group [102]. As the trial had to be stopped due to safety concerns, the study became underpowered to detect a treatment benefit of chloroquine [102]. An open-label clinical trial was undertaken in China on 150 SARS-CoV-2 infected patients with mild to moderate symptoms. Patients were either randomised to the normal standard of care or treated with hydroxychloroquine at 1200 mg daily for three days followed by a maintenance dose of 800 mg daily for another 2–3 weeks depending on severity [103]. There was no detectable difference in virus-negative conversion, although there was a greater report of adverse effects in the hydroxychloroquine treated patients [103]. There is currently no evidence to show any significant benefit of hydroxychloroquine or chloroquine in SARS-CoV-2 infection and high doses of the drugs may be associated with adverse effects. One explanation for the discrepancy between in vitro and in vivo findings may be due to the use of Vero cells for all in vitro experiments performed with SARS-CoV and SARS-CoV-2, which do not produce type I interferons [92,93,94,96,97,98].

In summary, despite various attempts to utilise hydroxychloroquine and chloroquine to control viral infections, they have not demonstrated clinical efficacy in treating any acute viral infections to date, either as a primary or adjunctive therapy. On the contrary, the immunomodulatory effects of hydroxychloroquine and chloroquine at in vivo biological concentrations may be exploited to enhance viral transduction during gene therapy.

## 4. Application of Hydroxychloroquine to Viral Vector-Mediated Retinal Gene Therapy

### 4.1. Immune Responses to AAV-Mediated Retinal Gene Therapy

AAV vectors have been widely utilised in gene therapy to treat a range of retinal genetic diseases due to their broad tissue tropisms, low pathogenicity, and relative low immunogenicity compared to other major viral vectors, such as lentiviruses and adenoviruses. Despite assumptions of immune privilege in the eye and low immunogenicity of AAV, inflammatory responses have been observed following subretinal injections of AAV. For instance, AAV2- and AAV8-injected cynomolgus macaques demonstrated a dose-dependent increase in capsid-specific neutralising antibodies and a systemic T cell response to the GFP transgene in two of the 14 animals that received high vector doses (1 × 10^11^ vector genomes (vg)/eye) [104]. Subretinal injection of cynomolgus macaques with an AAV2tYF vector (with three capsid tyrosine to phenylalanine mutations) expressing the gene *CNGB3* led to inflammatory responses in the anterior and posterior segments at both 1.2 × 10^11^ and 1.2 × 10^12^ vg/eye, with one animal developing severe endophthalmitis; all animals had an increase in neutralising antibodies to the AAV2tYF capsid [105]. Cynomolgous macaques injected with the AAV7m8 demonstrated high expression of glial fibrillary acidic protein (GFAP) (a marker for glial activation) at the highest vector dose (1 × 10^12^ vg/eye) [106]. Severe retinal inflammation was detected with signs of lymphocytic retinal infiltrates, perivascular inflammation, loss of RPE, and chronic choroidal inflammation [106]. The presence of a dose-dependent inflammatory response to AAV2-mediated retinal gene therapy was first observed in humans in a phase 1/2 clinical trial treating *RPE65*-associated Leber congenital amaurosis where five of the eight high dose patients (1 × 10^12^ vg/eye) had signs of intraocular inflammation, such as anterior uveitis, mild vitritis, and optic disc swelling [15]. In a phase 1/2 clinical trial of AAV2-mediated gene therapy for choroideremia, one of 14 high dose patients (1 × 10^11^ vg/eye) developed intraocular inflammation in the form of vitritis, retinitis, and choroiditis, which was associated with reduced visual function [17]. Similarly, in a phase 1/2 trial of AAV8-mediated gene therapy for *RPGR*-associated X-linked retinitis pigmentosa, seven out of nine high dose patients (0.6–4 × 10^11^ vg/eye) developed steroid-responsive subretinal inflammation associated with transient changes in retinal sensitivity [14]. Typically, inflammatory responses have been controlled in retinal gene therapy trials using a course of systemic immunosuppression (e.g., oral corticosteroid). However, persistent immune responses against the AAV transgene may diminish the long-term durability of the treatment. Currently, it is uncertain whether intraocular inflammation following AAV-mediated retinal gene therapy is directed at the viral capsid, therapeutic cassette, or the transgene product itself. Characterising the exact nature of the immune responses to AAV and devising methods for overcoming these counterproductive responses have become of great interest as gene therapies go from proof-of-concept to clinical application.

Intramuscular injection of recombinant AAV in wildtype mice elicited TLR9-mediated activation of pDCs and a type I interferon response independent of AAV serotype, suggesting that the TLR9-Myd88 pathway was critical for the activation of a CD8^+^ T cell response against the AAV capsid and transgene [107]. The immunogenic serotype AAVrh32.33 has also been shown to elicit a TLR9-mediated CD8^+^ T cell response in vivo, which was significantly diminished upon depletion of CpG sequences (the ligands for TLR9 receptors) in the viral genome [108]. The activation of adaptive immunity against AAV could lead to the production of transgene-specific antibodies, reduced transgene expression, and diminished durability of gene therapy. For instance, in a gene therapy trial for haemophilia B, an AAV serotype 2 vector containing the human Factor IX transgene delivered via hepatic artery infusion, led to a transient therapeutic level of transgene expression lasting 8 weeks due to destruction of transduced hepatocytes by an AAV capsid-specific CD8^+^ T cell response [109]. While AAVs containing self-complementary genomes can provide higher levels of transgene expression in vitro, they are associated with greater pro-inflammatory innate immune responses in vivo as the double-stranded AAV genome can activate TLR9 more strongly than ssDNA in standard AAV vectors [110]. Furthermore, we found that mouse embryonic fibroblasts lacking cGAS, which is capable of sensing secondary structures of ssDNA such as the AAV ITRs, showed significant increases AAV transgene expression compared with wildtype cells, suggesting that nucleic acid-sensing innate immune responses may be a key limiting factor for AAV transduction [111]. Together, these studies reveal the ability for AAV, similar to other viruses, to activate PRRs and elicit both intracellular and cellular immune responses that can determine the efficacy of gene therapy.

Key anti-viral sensors, including TLR9 and cGAS, and effectors, including IFN-γ, TNF-α, and IL-1β are upregulated in the retina following AAV gene therapy in vivo, in wildtype mice and non-human primates [111,112,113]. Release of pro-inflammatory cytokines within the retina can lead to increased permeability of the blood-retina barrier and enable infiltration of circulating leukocytes into the normally “immune-privileged” retinal environment [114,115,116]. This is supported by positive retinal immunohistochemical staining for MHC class I and II (suggestive of active antigen presentation) and CD8 (indicative of infiltrating cytotoxic T cells) following subretinal injection of AAV8 in non-human primates [112]. One possible source of retinal inflammation following AAV gene therapy may be related to the activation of retinal microglia, which are resident macrophage-like cells capable of detecting viral infections and orchestrating local immune responses through the activation of TLR9 [117] and cGAS [118], and recruitment of infiltrating leukocytes [119]. This would be consistent with increased immunohistochemical staining for ionized calcium-binding adaptor molecule 1 (IBA1) (a microglia activation marker) in the retina following subretinal injection of AAV in mice [112,113].

### 4.2. Improving the Efficacy of Retinal Gene Therapy

Since hydroxychloroquine and chloroquine are inhibitors of the anti-viral PRRs TLR9 and cGAS, they may provide a means of improving AAV transduction by modulating anti-viral responses during gene therapy. We tested this hypothesis by examining the effect of hydroxychloroquine on AAV2 transduction in vitro at a range of concentrations and found a significant increase in transgene expression at around 19 μM of hydroxychloroquine [111]. Similar enhancing effects on AAV transduction were observed in primary non-human primate retinal pigment epithelium (RPE) cells and human retina explants [111]. Co-administration of AAV vector and 19 μM hydroxychloroquine via subretinal injection in mice led to improved transgene expression compared with vector alone using both an AAV2 vector with a ubiquitous CAG promoter (4.6-fold improvement) and an AAV8(Y733F) vector with a photoreceptor-specific promoter (GRK1) (5.9-fold improvement) [111]. However, additional in vivo studies with therapeutic vectors and different AAV doses would be beneficial to further investigate the efficacy of hydroxychloroquine in retinal gene therapy.

The suppression of anti-viral pathways by hydroxychloroquine or chloroquine may ultimately prevent expression of downstream pro-inflammatory cytokines and recruitment of infiltrating leukocytes to the retina following AAV gene therapy. In addition, the cytokine IL-6, which has been shown to be inhibited by hydroxychloroquine and chloroquine, can induce B cell maturation and antibody production [42]. This may be of particular concern in patients seronegative for anti-AAV antibodies where AAV gene therapy to the first eye may lead to B cell maturation and the production of anti-AAV antibodies that could diminish the efficacy of repeat treatment to the second eye at a later date. Although AAV2 capsid-specific antibodies and T cell responses have been seen in animal models following readministration via the subretinal route [120], no such reaction was seen in a trial with 11 patients [121]. Nonetheless, transduction of the second eye did not seem to be significantly affected in either cases [120,121]. On the other hand, intravitreal administration of AAV appears to induce a significantly greater level of anti-AAV antibody response [122] and is more prone to antibody-mediated reduction of AAV transduction over repeated treatments [123]. This may be because of increased vector shedding in intravitreal AAV delivery [124], while the blood-retina barrier may limit exposure to circulating antibodies and immune cells during subretinal injections. Therefore, adjunctive therapy with hydroxychloroquine might be beneficial in intravitreally delivered retinal gene therapies by reducing the level of AAV neutralising antibody production to facilitate second eye treatment. Alternatively, the endopeptidase imlifidase (IdeS) has been developed as a means of eliminating anti-AAV antibodies [125]. Cynomologus macaques with pre-existing anti-AAV8 antibodies were intravenously injected with IdeS 24 h prior to AAV8-hFIX injections. This led to decreased titres of AAV8-specific IgG and neutralising antibodies and a significant increase in hFIX transgene expression. IdeS also enabled readministration of the novel AAV variant AAV-LK03 in African green monkeys, leading to a significant increase in transgene expression [125]. It remains to be seen whether such methods would prove effective in retinal gene therapy given that neutralising antibody responses are minimal following subretinal AAV injections [17]. Nonetheless, anti-AAV antibodies have been detected in some retinal gene therapy clinical trials following subretinal [15] and intravitreal injections [126,127], thus further work is necessary to determine the effect of retinal gene therapy on neutralising antibody responses.

Corticosteroids provide an additional means of controlling AAV-mediated inflammation and have been widely used in retinal gene therapy trials to control inflammatory responses [11,14,17]. Corticosteroid activity exists at the apex of an extensive signalling pathway that blocks several downstream inflammatory responses. For instance, by inducing expression of the anti-inflammatory molecule mitogen-activated protein kinase (MAPK) phosphatase 1; preventing transcriptional activity of nuclear factor kappa-light-chain-enhancer of activated B cells (NF-κB) that regulates downstream transcription of a vast range of cytokines; and blocking c-Jun–mediated transcription of innate immune factors [128]. However, despite this significant anti-inflammatory activity, little is known about the effect of corticosteroids on the efficacy of retinal gene therapy. In vivo studies to investigate the level of retinal transduction with and without corticosteroid treatment would thus be of significant interest. Further work is therefore needed to analyse the mechanism of the AAV-mediated immune responses and how these may be modified using adjunctive treatments during gene therapy.

## 5. Safety and Delivery of Hydroxychloroquine in Retinal Gene Therapy

### 5.1. Hydroxychloroquine Retinopathy

Although the first cases of retinopathy associated with chloroquine therapy were described in the late 1950s [129], retinal toxicity was considered infrequent until the turn of the century as only patients with symptoms of hydroxychloroquine retinopathy were detected [130]. The recognition of an increased risk of retinopathy in chloroquine users, as compared to hydroxychloroquine users, led to the reduction in the use of chloroquine across its treatment indications [131]. However, modern retinal imaging techniques, such as optical coherence tomography (OCT) and fundus autofluorescence, over the past 20 years have enabled the detection of pre-symptomatic disease [132]. Using such techniques, a major case-control study identified the prevalence of hydroxychloroquine retinopathy at 7.5% of patients taking the drug for more than 5 years, increasing to 20–50% after 20 years of therapy [133]. The risk factors for retinopathy included daily dose, duration of therapy, renal impairment, and concurrent tamoxifen therapy [133]. A daily dose of >5 mg/kg/day over a prolonged period was considered to confer a greater risk of hydroxychloroquine retinopathy [133], and safe dosing guidelines for chronic use of hydroxychloroquine were defined by this threshold [134]. Due to the risk of hydroxychloroquine retinopathy following chronic use, screening programmes were recommended in several countries to reduce the risk of permanent visual loss in this group [135,136]. It is important to note that clinically, hydroxychloroquine and chloroquine are not bioequivalent, and different daily doses are needed to achieve similar therapeutic effects. The safe limits of daily dosing with regards to retinopathy also differ, with 5 mg/kg/day for hydroxychloroquine and 2.3 mg/kg/day for chloroquine [136].

### 5.2. Routes of Administration

The adjuvant use of hydroxychloroquine to augment the effects of gene therapy at the time of AAV vector delivery requires some consideration of the route of delivery of hydroxychloroquine, the dose administered and the duration of therapy. The safe limits of orally administered hydroxychloroquine have been defined over the past 60 years. Although there is no identified safe dose which confers no risk, retinopathy is unlikely in the first 5 years of therapy unless concomitant risk factors are present [133]. Very high doses of hydroxychloroquine (1000 mg/day) used in clinical trials in oncology, where hydroxychloroquine is used as an inhibitor of autophagy, have resulted in retinopathy in two out of seven patients at 15 and 25 months, respectively [137,138]. Although the mechanism of retinal toxicity of systemically administered hydroxychloroquine is unknown, the dosing characteristics which produce retinopathy have been well described. In the context of retinal gene therapy, it is unlikely that short courses of orally administered hydroxychloroquine in the peri-operative period will have any detectable effects on retinal structure or function, even at a high daily dose of 1000 mg/day.

Although the systemic adjunctive use of hydroxychloroquine in retinal gene therapy has not yet been tested, this approach may be problematic given what is known about the pharmacokinetics of the drug. Hydroxychloroquine has a relatively long plasma half-life of 32 days, and elimination half-life in blood is approximately 50 days. The drug is highly sequestered in tissues, and therefore a steady-state plasma concentration is only achieved after 6 months of daily dosing [139]. Moreover, a study evaluating serum hydroxychloroquine levels in different individuals identified significant variability between subjects [140]. Thus, a consistent and predictable tissue concentration of hydroxychloroquine within the retina to enhance AAV retinal gene therapy would be difficult to achieve over a short interval. Furthermore, given that the effects of hydroxychloroquine on the intracellular innate immune response appear highly dose-dependent, this variability in tissue drug concentrations may have a significant influence on retinal photoreceptor or RPE transduction. A greater variability in clinical outcome measures might, therefore, be expected when compared to locally delivered hydroxychloroquine. Rabbit models have demonstrated the sequestration of systemically administered hydroxychloroquine within the pigmented tissues of the eye, such as the RPE, choroid, and ciliary body, where it is bound to melanin [141]. It is unclear how the immunomodulatory effects of hydroxychloroquine may influence the relative efficiency of transduction of AAV-delivered transgenes to the RPE or photoreceptors given this RPE-predominant tissue distribution from systemically administered hydroxychloroquine. Although the effects of systemically administered hydroxychloroquine have not been evaluated in any AAV gene therapy studies, it is likely that hydroxychloroquine would need to be administered in the weeks prior to AAV delivery to allow hydroxychloroquine to reach appropriate tissue concentrations within the eye. Both the efficacy of systemic hydroxychloroquine in the context of retinal gene therapy and tissue concentrations of hydroxychloroquine within the retina and RPE require further evaluation.

**Figure 3:**
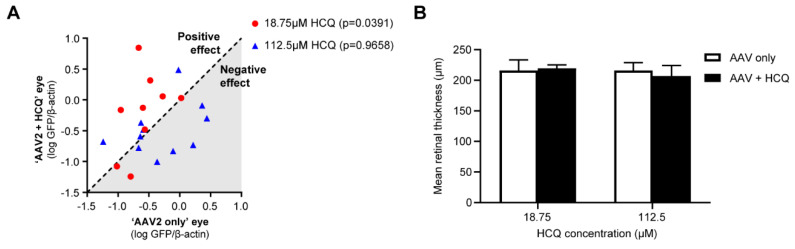
Dose effect of HCQ on improving the efficacy of adeno-associated viral (AAV)-mediated retinal gene therapy in vivo. C57BL/6J mice were subretinally injected with 1 × 10^8^ vector genomes of AAV8(Y733F) GRK1.GFP with and without either 18.75 μM (*n* = 10) or 112.5 μM (*n* = 11) HCQ. (**A**) The protein quantification of GFP expression normalised to β-actin (expressed as log_10_) 6 weeks post-injection of AAV only injected eyes (*x*-axis) plotted against AAV with HCQ injected eyes (*y*-axis). Each point represents an individual animal. Points above the line represent a positive effect and below a negative. The p-value for analysis between paired eyes is given in the legend using a Wilcoxon matched-pairs signed rank test. (**B**) Mean total retinal thickness measured by in vivo spectral domain optical coherence tomography imaging (± SEM).

The delivery of hydroxychloroquine by subretinal injection allows for precise control over the local drug concentration. In 2013, an in vitro study used chloroquine at 100 μM as a tool for alkalising endosomes, a concentration known to raise the pH of these compartments, and demonstrated a decrease in AAV transduction in a hepatocellular carcinoma cell line (HepG2) [142]. In contrast, we showed that 19 μM hydroxychloroquine enhanced AAV-mediated transgene expression in vivo by around 5-fold [111]. The lower concentration of hydroxychloroquine used would primarily be expected to have an immunomodulatory effect and minimal influence on endosomal pH. When comparing the effects of low (19 μM) versus high (113 μM) dose of hydroxychloroquine co-administered subretinally with AAV in vivo, we found that 19 μM hydroxychloroquine led to significantly increased transgene expression, while 113 μM hydroxychloroquine had no effect or in some cases a negative effect on AAV transduction (Figure 3A). Moreover, no retinal structural change was detected by OCT with this single low dose of hydroxychloroquine (Figure 3B) [111]. Given that hydroxychloroquine was only delivered as a one-off treatment together with the AAV vector, the toxic effects associated with prolonged hydroxychloroquine treatment are unlikely. However, long-term assessment of retinal function using an electroretinogram would be required to fully investigate the safety of subretinally delivering hydroxychloroquine. Taken together, these findings are consistent with the hypothesis that a low concentration of locally delivered hydroxychloroquine could improve viral transgene expression by inhibiting PRR-mediated anti-viral responses, while high concentrations of hydroxychloroquine may impair viral entry by altering endosomal pH.

### 5.3. Potential Clinical Applications

Current AAV retinal gene therapy trials and therapeutic protocols, in the case of voretigene neparvovec for *RPE65*-associated Leber congenital amaurosis, generally include a perioperative period of systemic immunosuppression with prednisolone to reduce the risk of retinal inflammation [11,14,17]. Nevertheless, at high vector doses cases of intraocular inflammation have been observed requiring supplementary corticosteroid treatment. This can include oral prednisolone, dexamethasone eye drops, and intravitreal dexamethasone implants (Ozurdex), as demonstrated in the phase 1/2 dose-escalation gene therapy trial for X-linked retinitis pigmentosa [14]. Corticosteroids provide an effective means of controlling ocular inflammation; however, systemic corticosteroid usage may be associated with a range of potential adverse effects, including activation of viral retinitis in previously immunocompetent patients [143,144]. Intraocular or periocular corticosteroid use may also be associated with an increased risk of acute retinal necrosis secondary to HSV [145,146]. Since the dose of hydroxychloroquine administered in the sub-retinal space in AAV gene therapy potentiates viral action, there is a theoretical risk of viral retinitis. However, acute retinal necrosis has not been reported in long-term systemic hydroxychloroquine users despite clear evidence for drug accumulation within the RPE. Hydroxychloroquine in the context of subretinal delivery may, therefore, function as an immunomodulatory rather than immunosuppressive agent. This may suggest that subretinal administration of a single low dose of hydroxychloroquine as an adjuvant to AAV gene therapy is of low risk while offering the potential to reduce the AAV dose required, thus reducing the risk of treatment-induced retinal inflammation and the need for systemic steroids to counter this response. However, while existing evidence supports the safety of low dose hydroxychloroquine in healthy retinae, it is unclear whether the degenerate RPE and photoreceptors in inherited retinal dystrophies may respond differently to the same concentration of hydroxychloroquine.

## 6. Conclusions

An important feature of the mechanism of action of HCQ and CQ is their ability to accumulate in intracellular compartments. However, the activity of these drugs within acidic vesicles may largely rely on their concentration. High doses of HCQ and CQ can alkalise endosomes and lysosomes to impair their function, while low doses appear to have minimal effects on pH but can prevent activation of intracellular PRRs to modulate downstream innate immune responses. This dual activity may explain the contradictory effects of HCQ and CQ seen in viral infections, with low concentrations providing potential to minimise anti-viral responses. AAV gene therapy is a promising treatment for inherited retinal disease; however, clinical efficacy is limited by the proportion of target cells that successfully express the therapeutic transgene. Adjunctive use of HCQ provides a means of inhibiting restrictive anti-viral intracellular immune responses to improve transgene expression and enhance the therapeutic effect to reach the transduction threshold needed to prevent disease progression. Given the risk of deleterious tissue inflammation with high doses of AAV, HCQ may also enable the use of lower and safer vector doses to achieve a given treatment effect. When applied to viral infections in general, it remains to be seen whether an optimal dosage of HCQ may be found to reduce unwanted tissue inflammation due to anti-viral immune responses in order to reduce overall disease severity or duration.

## Figures and Tables

**Table 1:** Effects of hydroxychloroquine and chloroquine on selected viruses in vitro.

Effect	Drug	Concentration (μM)	Virus	Viral Genome	Cells	Reference
Inhibition of viral replication ^1^	CQ	75	Yellow fever virus	ssRNA	P388D1	[85]
		100	Semliki Forest virus	ssRNA	BHK-21	[62]
		10–100	Hepatitis A virus	ssRNA	BS-C-1	[64]
		150	HSV-1	dsDNA	HuH7	[68]
		250–4000	Varicella zoster virus	dsDNA	Mononuclear cells	[86]
	HCQ	1–1000	HIV-1	ssRNA	T cell and macrophage hybridoma cell line	[67]
Inhibition of virus-mediated immune response ^1^	CQ	10 and 100	Hepatitis C virus	ssRNA	Huh7 and macrophages	[76,77]
		10	HSV-2	dsDNA	pDCs	[80]
		1–100	Vesicular stomatitis virus	ssRNA	pDCs	[79]
		10	Influenza A virus	ssRNA	pDCs	[78]
		5 and 100	HIV-1	ssRNA	pDCs and PBMCs	[73,74]
	HCQ	10 and 20	Epstein Barr virus	dsDNA	pDCs and monocytes	[81,82]

^1^ In vitro studies conducted in Vero cells were excluded because of the inability for these cells to produce type I interferon responses, making them unsuitable for assessing the immunomodulatory effects of HCQ and CQ. HSV, herpes simplex virus; pDC, plasmacytoid dendritic cell; HIV, human immunodeficiency virus.

## Abbreviations

AAV	Adeno-associated virus
HCQ	Hydroxychloroquine
CQ	Chloroquine
ssDNA	Single-stranded DNA
ITR	Inverted terminal repeat
HIV	Human immunodeficiency virus
TLR	Toll-like receptor
PRR	Pattern recognition receptor
dsRNA	Double-stranded RNA
ssRNA	Single-stranded RNA
ODN	Oligodeoxynucleotide
PBMC	Peripheral blood mononuclear cell
cGAS	Cyclic GMP-AMP synthase
IFN	Interferon
TNF	Tumour necrosis factor
IL	Interleukin
ISG	Interferon-stimulated gene
MHC	Major histocompatibility complex
HSV	Herpes simplex virus
SARS-CoV	Severe acute respiratory syndrome coronavirus
pDC	Plasmacytoid dendritic cell
vg	Vector genomes
OCT	Optical coherence tomography
